# An Analysis of Culling Patterns during the Breeding Cycle and Lifetime Production from the Aspect of Culling Reasons for Gilts and Sows in Southwest China

**DOI:** 10.3390/ani9040160

**Published:** 2019-04-12

**Authors:** Chao Wang, Yinghui Wu, Dingming Shu, Hongkui Wei, Yuanfei Zhou, Jian Peng

**Affiliations:** 1 Department of Animal Nutrition and Feed Science, College of Animal Science and Technology, Huazhong Agricultural University, Wuhan 430070, China; wangchao1028@163.com (C.W.); academy_wuyinghui@163.com (Y.W.); weihongkui@mail.hzau.edu.cn (H.W.); zhouyuanfei@mail.hzau.edu.cn (Y.Z.); 2 Sichuan Dekang Agriculture and Animal Husbandry Technology co. LTD, Chengdu 610000, China; sdm_028@163.com; 3 The Cooperative Innovation Center for Sustainable Pig Production, Wuhan 430070, China

**Keywords:** culling patterns, gilt, lifetime production, sow

## Abstract

**Simple Summary:**

Unplanned removal of gilts and sows shortens the longevity and decreases the production efficiency of commercial herds. A comparison between sow culling studies is problematic due to differences in genetics, housing conditions, and feeding and management among different countries. Analyzing and monitoring culling patterns in sows in a particular area could help improve domestic feeding and the managing level. Based on the large sample of production data, we found that disease, lameness, return to estrus, and anestrus beyond seven days sharply decreased lifetime production in sows. We suggest that producers take effective measures to reduce anestrus beyond nine months, reproductive system disease, return to estrus, and low or no milk production for gilts, weaned sows, gestating sows, and lactating sows, respectively. We also recommend producers pay more attention to disease, lameness, and return to estrus for sows at low parity.

**Abstract:**

To investigate culling patterns during the breeding cycle and lifetime production associated with culling reasons, 19,471 culling records were collected in southwest China. Lifetime pigs born alive (LPBA) and parity for culling reasons, and reason distribution at different parities and breeding cycle were analyzed. Sows culled for stress and death (SD), lameness (LA), common disease (CD), not being pregnant, return to estrus, and abortion (NP) had fewer than 20 LPBA (*p* < 0.05). Gilts were mainly culled for anestrus beyond nine months (AB9), CD, and LA, while weaned sows were culled for reproductive system disease (RS), CD, and anestrus beyond seven days (*p* < 0.0033). Gestating sows were mainly culled for NP, CD, and SD, while lactating sows were mainly culled for low or no milk production (NM), poor litter size, and CD (*p* < 0.0033). Moreover, sows were mainly culled at parity 0, 1, and 2 (*p* < 0.0024). Besides CD and RS, LA and NP were the primary reasons for parity 1 and 2 culls, respectively. In conclusion, SD, LA, CD, and NP sharply decrease sow lifetime production. AB9, RS, NP, and NM mainly occurred in gilts, weaned, gestating, and lactating sows, respectively. Low parity sows had a higher risk of CD, RS, LA, and NP.

## 1. Introduction

A proper culling policy in commercial herds is critical for the reproductive profile and financial performance of breeding animals [1,2]. Lifetime production of sows can be measured as lifetime pigs born alive (LPBA), and it is affected by sow culling reasons [3,4]. Inappropriate culling decreases lifetime production of sows and places a major financial burden on producers [5]. Therefore, analyzing and monitoring culling patterns is conducive to administrative decisions on productivity and economic results of breeding herds.

Annual culling rates on pig farms range from 35.7 to 49.5% in the USA, Spain, Sweden, and Japan [6,7]. Reproductive problems and lameness were the major culling reasons in some studies, but these did not distinguish between the stages of the breeding cycle in sows [4,8,9]. The risk of removing a breeding female is not the same throughout its life [10], and it may vary among countries, herds, and parities [1]. Furthermore, lifetime production in culled females varies with culling reasons [4,11], while lifetime production for individual females associated with different culling reasons has not been fully elucidated.

Southwest China is becoming one of the main pork-producing and sow-breeding regions of the country. The present study aimed to analyze (1) lifetime production of sows from the aspect of different culling reasons; (2) and distribution of culling reasons for gilts and sows during different parities and stages of the breeding cycle in southwest China.

## 2. Materials and Methods

Animal Care and Use Committee approval was not obtained for this study because the data were obtained from an existing database of a single large integrated pork production company.

### 2.1. Herds, Animals, and Management

This study was conducted in 24 breeding and commercial herds overseen by a single large integrated pork production company, which was located in southwest China. The inventory of each farm comprised of 2000 to 6000 breeding sows, and nearly 100,000 gilts and breeding sows in total were raised in the company. The culling rate of these pig farms approximated 35%, with a fluctuation ranging from 30% to 40%. Gilts and sows included in this study were purebred Landrace, Yorkshire, Duroc, Landrace x Yorkshire, and Yorkshire x Landrace, and the numbers of them were 3612, 5696, 171, 9351, and 641, respectively. All herds in the present study performed similar procedures in mating, farrowing, and culling. However, each regional herd was an independent business unit with independent technicians developing their own implementation standards. The technicians of these regional pig herds referred to and selected the headquarters guidelines of the company to form a technical implementation standard with regional differentiation based on the production and operation requirements in their regions. Gestating houses were equipped with an automatic feeding system (Automated Production Systems, Assumption, IL, USA), and sows were housed individually in stalls with slatted flooring. Gestating sows were moved from gestating stalls to farrowing crates on day 106 ± 1 of gestation. Lactating sows, whose lactation length was 18 to 21 days, were fed four times every day. The amount of feed for gestating and lactating sows was 2.8 kg/day and 5.5 kg/day, respectively. The temperatures of the gestation and farrowing house were maintained at 18 °C to 21 °C and 20 °C to 22 °C, respectively. Detection of estrus in sows using boar contact was performed twice a day after weaning. All herds were artificially inseminated at 12 h and 36 h after first detecting estrus, and a real-time ultrasound was used to detect pregnancy 23 to 28 days after insemination.

### 2.2. Data Collection and Classification of Culling Reasons

The JD Edwards software (Sichuan Dekang Agriculture and Animal Husbandry Technology Co. LTD, Chengdu, China) was used to record and store data on all breeding and commercial herds. Producers of these herds were requested to submit their initial culling data of gilts and sows (from January 2016 to December 2017) to the College of Animal Science and Technology, Huazhong Agricultural University. The following information associated with culling was processed: Tag number, parity at culling, and culling reason. Culling reason in this study was divided into: (1) Common disease (CD; mainly including diseases that cause fever and loss of appetite); (2) reproductive system disease (RS); (3) not pregnant, return to estrus, and abortions (NP); (4) lameness (LA); (5) anestrus beyond seven days for sows (AB7); (6) anestrus beyond nine months for gilts (AB9); (7) stress and death (SD; sows suffering from stress exhibited symptoms of muscle tremors, flushed skin, and shortness of breath); (8) poor litter size (PL); (9) low or no milk production (NM); (10) old age (OA); and (11) others (OTH; mainly including failure to service, farrowing difficulties, poor maternal behavior, abnormal estrus, low effectiveness of nipple, and abnormal pudendum). To calculate LPBA, breeding and farrowing records for each sow before culling were collected for analysis.

### 2.3. Statistical Analysis

All analytical procedures were performed in SAS software (version 9.4; SAS Inst. Inc. Cary, NC, USA). Data of parity at culling and LPBA were presented as least square mean and standard error and analyzed using a mixed linear model (PROC MIXED) with culling reason as the fixed effect and farm and year as random effects, respectively. Particularly, culling reason AB9 was excluded from the mixed model because AB9 was only a reason for gilt culling. When significant treatment differences were detected, the PDIFF option of the LSMEANS statement of the MIXED procedure was used to compare individual least square means. For the examination of data of removal reason distribution at different parities and stages of the breeding cycle, a chi-square test (PROC FREQ) was performed with the significance level at 5%. Litter size, milk production, old age, and problems with returning to estrus were excluded from the analysis of reason distribution at different parities for gilts. Then, the significance level had to be adjusted as follows for the comparison between any two culling reasons, to reduce the probability of a class I error when overall difference was significant.

(1) $$\alpha^{\prime }=\frac{2\alpha }{R*\left(R-1\right)},$$

where α’ represented the adjusted significance level. R represented the number of removal reasons (groups).

## 3. Results

### 3.1. Reasons, Parities, and LPBA of Culling Gilts and Sows

As shown in Table 1, we identified 11 subgroups of gilt and sow culling reasons, which belonged to planned and unplanned types in this study. Less than 2% of culls was planned and more than 98% was unplanned for gilts and sows. The average culling parity and LPBA of all culls were 2.27 and 18.65, respectively, and unplanned culls had a lower culling parity and LPBA than planned culls (*p* < 0.05). Furthermore, sows culled for SD (4.93%), LA (10.53%), CD (23.70%), and NP (7.91%) had less than 20 LPBA (*p* < 0.05).

### 3.2. Culling Reasons for Gilts and Sows during Different Stages of the Breeding Cycle

**Figure 1:**
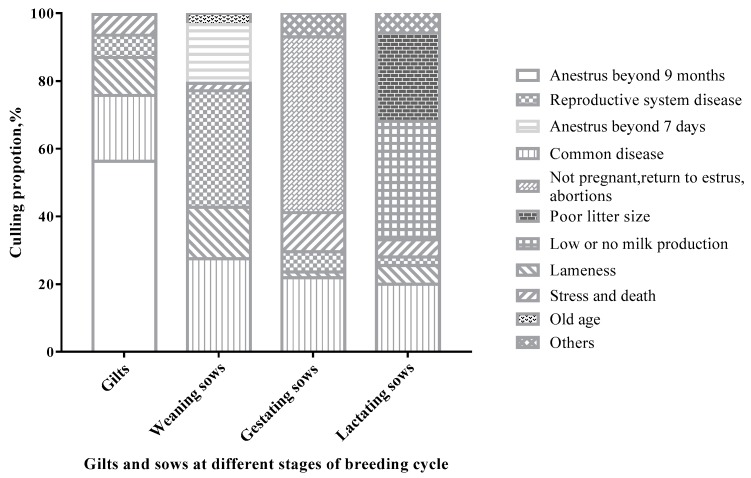
Culling reasons distribution in gilts and sows at different stages of the breeding cycle (gilts = 3685; weaned sows = 9079; gestating sows = 2967; lactating sows = 3740). The difference between the proportion of reproductive system disease and stress and death for gilts, others, and reproductive system disease for gestating sows was not significant (*p* > 0.05). In addition, the difference between the proportion of others and lameness, others and stress and death, lameness and stress and death for lactating sows was also not significant (*p* > 0.05). In addition to the above, the difference between any two culling reasons for gilts, gestating sows (*p* < 0.0033), weaned sows, and lactating sows (*p* < 0.0024) was significant.

As shown in Figure 1, AB9 (56.31%), CD (19.46%), and LA (11.21%) were the three major reasons for gilt culling (*p* < 0.0033). Regarding weaned sows, RS (34.42%), CD (27.50%), AB7 (17.30%), and LA (15.22%) were the four main reasons for culling (*p* < 0.0024). For gestating sows, NP (51.94%), CD (21.94%), and SD (11.56%) were the three major reasons for culling (*p* < 0.0033). In addition, NM (34.95%), PL (26.04%), and CD (20.03%) were the three main causes in culled lactating sows (*p* < 0.0024).

### 3.3. Parity Distribution of Different Reasons and Reasons Distribution of Different Parities for Gilt and Sow Culling

The results of parity distribution of different removal reasons and removal reason distribution of different parity sows are presented in Table 2. Parity 2 culling sows were the most prevalent, accounting for 22.53% of total culls, followed by parity 1 sows (20.30%), gilts (19.93%), parity 3 sows (13.05%), parity 4 sows (11.93%), parity 5 sows (8.24%), and sows with more than 6 parities (4.03%; *p* < 0.0024). AB9 and SD mainly occurred in gilts, while LA and OTH mainly occurred in sows culled at parity 1 (*p* < 0.0024). In addition, RS, CD, NP, AB7, and PL mainly occurred in sows culled at parity 2 (*p* < 0.0024). Regarding removal reason distribution of different parity sows, parity 1 and 2 sows were mainly culled due to CD and RS (*p* < 0.0011). Besides these two removal reasons, LA and NP were the most prevalent in sows culled at parity 1 and 2, respectively (*p* < 0.0011). Parity 3 sows were mainly culled for CD, RS, AB7, LA, NP, and NM (*p* < 0.0011).

## 4. Discussion

This study has been the first report to study the regulation of gilt and sow culling in southwest China, which is one of the key areas of pig industry development in the country. The results showed that the proportion of unplanned culls was over 98%, and parity at culling and LPBA of unplanned culls were sharply decreased compared to planned culls. Sows were mainly culled at low parities (parity 0, 1, and 2), and culling reasons for sows were different distribution at different parities and stages of the breeding cycle.

Some studies stated that annual culling rates fluctuated from 35.7 to 49.5% in commercial herds, which was a little bit higher than 35% in this study [6,7]. A possible reason for this may be the difference in management, feeding, genetics, climate, and housing conditions [1]. In this study, the proportion of unplanned sow culling was 98.44%, higher than 71.8% [6] and 78.1% [8]. The increased proportion of unplanned culls was due to the fact that only old age was considered as planned culling for sows in this study, while poor litter size, poor lactation and rearing ability, poor maternal behavior, and farrowing difficulties were included in planned culls in another study [8]. This means that there is much room for improvement in feeding and management of sow herds in southwest China. Common disease, RS, AB9, LA, AB7, and NP, which sharply decreased culling parity and LPBA, were the main reasons for gilt and sow culling in the present study. A proper culling policy in sow herds is a prerequisite to maintaining a stable parity profile of the breeding animals and consistent production [1,12]. Not being pregnant, a return to estrus, and abortion (NP) and LA decreased LPBA through expanding non-productive days [13] and shortening the longevity of culling sows in pig herds, respectively [14]. Sasaki and Koketsu [4] reported LPBA for sows culled due to NP (culling parity: 2.5) and LA (culling parity: 2.7) were 36.0 and 32.4, respectively, which are higher than those in the present study. Therefore, these culling reasons should arouse producer attention. Analyzing removal regulation of gilts and sows could help producers to reduce unplanned culls and improve sow lifetime production in China.

In this study, AB9 and CD were the two major reasons for gilt culling, while RS, CD, and AB7 were the main reasons for weaned sows. In addition, we found that gestating sows were mainly culled for NP and CD, while lactating sows were culled due to NM and PL. These results indicated that the culling policy for sows at different stages of the breeding cycle should be different in commercial herds [2]. Regarding parity at culling, a sow should achieve between 3 and 4 parities to reach a positive net present value and produce enough piglets to pay for herself [15,16]. However, the average parity of unplanned culls was 2.27 in southwest China, lower than that in other counties [6,11,17,18]. This was mainly due to more gilts and sows being culled at low parities (1 and 2; totally 62.76%), which is broadly in line with other studies [19,20,21]. Further analysis revealed that LA and NP were the most prevalent reasons for parity 1 and 2 culls besides CD and RS, respectively.

A better knowledge of reasons for culling can be beneficial in identifying underlying diseases or management problems in breeding herds [12]. Disease, which included CD and RS in this study, may be partly associated with poor management and hygiene practices in herds [22]. It was reported that a bacterial infection, such as *E. coli, Staphylococcus spp.* and *Streptococcus spp.*, was the major reason for genital inflammation in sows [23,24,25]. Therefore, strengthening hygiene management is essential for the prevention of certain diseases. With regard to AB9, inappropriate nutritional management during pre-pubertal periods, induction of puberty onset, and development procedures are highly associated with later puberty age [18]. A high age at first-service increases culling risk and leads to a low farrowing rate and late pregnancy loss in gilts [26,27]. Not being pregnant, a return to estrus, and abortion was the most prevalent removal reason for gestating sows. It is necessary for producers to perform frequent estrus checks for served sows in the first three to six weeks post service to minimize non-productive days [13]. In addition, excessive backfat before farrowing [28], a high ambient temperature during lactation [29,30], improper dietary energy source used during lactation [31], and disease reduce the feed intake and milk production of lactating sows. Avoiding the problems mentioned above may contribute to decreasing unplanned culls in commercial herds.

## 5. Conclusions

In conclusion, unplanned culling risks that mainly include CD, RS, AB9, LA, AB7, and NP were common in southwest China and reduced the parity at culling and LPBA of sows. Removal reasons AB9, RS, NP, and NM mainly occurred in gilts, weaned sows, gestating sows, and lactating sows, respectively. In addition, CD, RS, LA, and NP were the most prevalent reasons for sows culled at low parities. Therefore, producers should take parity and stage of the breeding cycle into consideration in case of sow culling. Since this study was an observational study using records from commercial herds, it provides valuable information on the knowledge of gilt and sow culling in southwest China.

## Figures and Tables

**Table 1:** The reasons, parities, and lifetime pigs born alive (LPBA) of culling gilts and sows.

Culling Reason	*n*	Culling Proportion	Parity at Culling		LPBA	
			Lsmeans ^1^	SE ^2^	Lsmeans	SE
Unplanned						
Anestrus beyond 9 months	2075	10.66%	0.00	0.00	0.00	0.00
Stress and death	959	4.93%	2.07 ^f^	0.05	16.22 ^g^	0.47
Lameness	2050	10.53%	2.04 ^f^	0.03	17.54 ^f^	0.32
Common disease	4614	23.70%	2.22 ^e^	0.02	17.95 ^ef^	0.21
Not pregnant, return to estrus, and abortions	1541	7.91%	2.67 ^c^	0.04	18.57 ^e^	0.37
Reproductive system disease	3636	18.67%	2.57 ^d^	0.03	21.09 ^d^	0.24
Others	440	2.26%	2.43 ^d^	0.07	22.39 ^d^	0.69
Anestrus beyond 7 days	1571	8.07%	2.67 ^c^	0.04	22.62 ^c^	0.36
Poor litter size	974	5.00%	3.25 ^b^	0.05	23.43 ^c^	0.46
Low or no milk production	1307	6.71%	3.21 ^b^	0.04	27.26 ^b^	0.40
Planned						
Old age	304	1.56%	5.72 ^a^	0.09	53.19 ^a^	0.83
Total	19,471	100.00%	2.27	0.12	18.65	0.11

^1^ Lsmeans within a column with different superscripts significantly differ (p < 0.05). ^2^ SE = stand error.

**Table 2:** Parity distribution of different reasons and reasons distribution of different parities for gilt and sow culling.

Culling Reasons	Parity at Culling						
	0	1	2	3	4	5	≥6
Reproductive system disease	6.63% ^e^	18.04% ^c^(19.47% ^b^) ^1^	26.84% ^a^(22.25% ^b^)	23.10% ^b^(25.31% ^a^)	14.91% ^d^(23.34% ^a^)	7.62% ^e^(17.26% ^b^)	2.86% ^f^(13.27% ^b^)
Common disease	15.54%^c^	20.74% ^b^(28.41% ^a^)	24.49% ^a^(25.76% ^a^)	17.75% ^c^(24.68% ^a^)	11.25% ^d^(22.35% ^a^)	7.82% ^e^(22.49% ^a^)	2.41% ^f^(14.16% ^b^)
Not pregnant, return to estrus, abortions	- ^2^	19.34% ^b^(8.85% ^e^)	35.95% ^a^(12.63% ^c^)	19.14% ^b^(8.89% ^b^)	13.30% ^c^(8.83% ^c^)	8.37% ^d^(8.04% ^c^)	3.89% ^e^(7.65% ^c^)
Lameness	20.15% ^b^	25.27% ^a^(15.38% ^c^)	19.07% ^b^(8.91% ^d^)	14.98% ^c^(9.25% ^b^)	10.59% ^d^(9.35% ^cd^)	6.93% ^e^(8.85% ^c^)	3.02% ^f^(7.91% ^c^)
Anestrus beyond 7 days	-	24.06% ^ab^(11.22% ^d^)	28.01% ^a^(10.03% ^d^)	20.24% ^b^(9.58% ^b^)	15.60% ^c^(10.55% ^c^)	8.53% ^d^(8.35% ^c^)	3.57% ^e^(7.14% ^c^)
Anestrus beyond 9 months	100.00%	-	-	-	-	-	-
Stress and death	23.57% ^a^	12.30% ^b^(3.50% ^gh^)	26.80% ^a^(5.86% ^e^)	16.37% ^b^(4.73% ^c^)	14.18% ^b^(5.86% ^d^)	5.21% ^c^(3.13% ^d^)	1.56% ^d^(1.91% ^d^)
Poor litter size	-	9.24% ^e^(2.67% ^h^)	28.23% ^a^(6.27% ^e^)	22.18% ^b^(6.51% ^c^)	18.79% ^bc^(7.88% ^cd^)	13.86% ^cd^(8.41% ^c^)	7.70% ^e^(9.57% ^bc^)
Low or no milk production	-	16.07% ^bc^(6.23% ^f^)	20.05% ^ab^(5.97% ^e^)	22.26% ^a^(8.77% ^b^)	17.60% ^ab^(9.91% ^c^)	16.45% ^bc^(13.40% ^b^)	7.58% ^d^(12.63% ^bc^)
Old age	-	-	-	-	-	37.50% ^b^(7.10% ^c^)	62.50% ^a^(24.23% ^a^)
Others	2.95% ^e^	32.73% ^a^(4.27% ^g^)	23.18% ^bc^(2.33% ^f^)	17.27% ^cd^(2.29% ^d^)	10.23% ^d^(1.94% ^e^)	10.91% ^d^(2.99% ^d^)	2.27% ^e^(1.53% ^d^)
Total	19.93% ^b^	20.30% ^b^	22.53% ^a^	13.05% ^c^	11.93% ^d^	8.24% ^e^	4.03% ^f^)

^1^ Numbers outside the brackets in a row are presented as the parity distribution of different reasons for gilt and sow culling (data within a row with different superscripts significantly differ (*p* < 0.0024)); numbers inside the brackets in a column are presented as the reasons distribution of different parities for gilt and sow culling (data within a column with different superscripts significantly differ (*p* < 0.0011)).^2^ ”-“ represented some of the removal reasons which were not included in certain parities for gilts and sows.
